# P-64. Variations in the treatment of Cutibacterium acnes Infections of the Bones and Joints and Associated Outcomes

**DOI:** 10.1093/ofid/ofae631.271

**Published:** 2025-01-29

**Authors:** Adrienne Elizabeth Yun, Ashish Bhargava, Susan M Szpunar, Leonard B Johnson

**Affiliations:** Ascension St. John, Franklin, Michigan; Ascension St. John Hospital; Wayne State University School of Medicine, Grosse Pointe Woods, Michigan; Ascension St. John Hospital, MI; Ascension St. John Hospital, MI

## Abstract

**Background:**

*Cutibacterium acnes* is an emerging pathogen in bone and joint infections. While treatment guidelines recommend the use of β-lactam therapy for *C. acnes* infections, the lack of routine susceptibility testing may result in wide variation in treatment regimens among physicians. We assessed the treatment regimens chosen by physicians for patients with bone and joint infections with *C. acnes* and the treatment outcomes of β-lactam and non-β-lactam regimens.

**Methods:**

This was a multi-center, historical cohort study comparing treatment regimens in patients diagnosed with *C. acnes* for both native and prosthetic bone and joint infection. The study included patients with monomicrobial cultures positive for *C. acnes* and diagnosed with septic arthritis, prosthetic joint infections, osteomyelitis, or discitis between 7/1/2018- 6/30/2023. Data collected included antimicrobial therapeutic regimens, surgical intervention, as well as treatment failure and success. Data were analyzed using chi square test, Student’s t-test, and the Mann-Whitney U test.

**Results:**

Among 49 included patients, 65.3% of infections resulted from prosthetic joint infection, 26.5% from osteomyelitis, and 8.2% from septic arthritis. The most common intravenous antibiotics chosen to treat *C. acnes* were ceftriaxone (51%), vancomycin (16.3%), and penicillin (6.1%) with a mean treatment duration of 43.4 days. The most common oral antibiotic treatment was doxycycline (20.4%), amoxicillin (18.4%), cephalexin/cefadroxil (12.2%) with a median outpatient treatment duration of 38 days (range 5, 364). Overall, there were no difference in treatment outcomes between patients treated with β-lactams vs, non-β-lactams at six months, including cure (58.8% vs. 71.4%, p=0.53), persistence of symptoms (26.5% vs. 14.3%, p=0.49), recurrence (17.6% vs. 28.5%, p=0.51), and requiring suppressive therapy (23.5% vs. 28.6%, p=0.78), respectively.

**Conclusion:**

Physicians preferred β-lactam antibiotics for treating patients with bone and joint infections caused by *C. acnes*. There were no differences in outcomes for treating *C. acnes* bone and joint infections between β-lactam and non-β-lactam antibiotic regimens. Further studies should be performed to confirm this observation.

**Disclosures:**

**All Authors**: No reported disclosures

